# Civil airline fare prediction with a multi-attribute dual-stage attention mechanism

**DOI:** 10.1007/s10489-021-02602-0

**Published:** 2021-08-03

**Authors:** Zhichao Zhao, Jinguo You, Guoyu Gan, Xiaowu Li, Jiaman Ding

**Affiliations:** 1 Faculty of Information Engineering and Automation, Kunming University of Science and Technology, Kunming, 650500 China; 2 Yunnan Key Laboratory of Artificial Intelligence, Kunming University of Science and Technology, Kunming, 650500 China

**Keywords:** Civil airline fare prediction, Time series, Attention mechanism, LSTM

## Abstract

Airfare price prediction is one of the core facilities of the decision support system in civil aviation, which includes departure time, days of purchase in advance and flight airline. The traditional airfare price prediction system is limited by the nonlinear interrelationship of multiple factors and fails to deal with the impact of different time steps, resulting in low prediction accuracy. To address these challenges, this paper proposes a novel civil airline fare prediction system with a Multi-Attribute Dual-stage Attention (MADA) mechanism integrating different types of data extracted from the same dimension. In this method, the Seq2Seq model is used to add attention mechanisms to both the encoder and the decoder. The encoder attention mechanism extracts multi-attribute data from time series, which are optimized and filtered by the temporal attention mechanism in the decoder to capture the complex time dependence of the ticket price sequence. Extensive experiments with actual civil aviation data sets were performed, and the results suggested that MADA outperforms airfare prediction models based on the Auto-Regressive Integrated Moving Average (ARIMA), random forest, or deep learning models in MSE, RMSE, and MAE indicators. And from the results of a large amount of experimental data, it is proven that the prediction results of the MADA model proposed in this paper on different routes are at least 2.3% better than the other compared models.

## Introduction

Air travels are becoming more and more popular in China, and numerous online booking channels for aircraft tickets are now available. It is well-recognized that airlines make decisions about aircraft ticket prices based on the time of purchase. Airlines nowadays use complex strategies to dynamically allocate ticket prices, and these strategies take into account a variety of financial, marketing, commercial, and social factors. Because of the high complexity of the pricing model and the dynamic price changes, it is tricky for customers to buy tickets at the lowest price. Therefore, several applications have been developed recently to predict the ticket price, thereby guiding customers to buy tickets at the most appropriate time. Specifically, Hopper [23] is a relatively mature airfare forecast app, producing an accuracy of 95%, and 60% of its push messages tell its consumers that it is not the optimal time to order tickets yet.

Ticket price forecasts are of great reference value for the aviation industry. Ticket prices are determined by various factors, such as airlines, days of early purchase, as well as the departure time and airport. Airlines can adjust their ticket prices based on these factors to get the expected income for an effective pricing strategy [10, 19, 30].

However, several factors can limit the accuracy of air ticket price forecasts. First of all, the price of air tickets is a random walk time series, which is affected by the purchase time and other related factors; Secondly, with the ARIMA model, only simple non-stationarity type relationships can be acquired, but predictions of conventional time series are non-linear and non-stationary. The time series data used for prediction is generally required as regressive and periodic, which is not the case with air ticket price forecasts. Finally, ticket prices are affected by many uncertain factors, such as the long-term impact from governmental regulations, the short-term impact from the market and the weather, as well as some unexpected or international events. One example of such events is the novel coronavirus outbreak, which led the entire international airline industry to experience a downturn.

A linear quantile model [18] was proposed to predict the ticket prices in 2014. The model integrates four LR models to obtain the best fitting effect, mainly to provide passengers with unbiased information about whether to purchase tickets or wait longer to provide better prices. Besides, Tziridis et al. [28] used eight machine learning models to predict ticket prices, including ANNs, RF, SVM, and LR, and compared their results. Bagging Regression Tree was found to be the best model in their comparison, as it is stable and unaffected by various input feature sets. Moreover, deep learning has also demonstrated great promise and made significant progress in computer vision and natural language processing. Neural networks [1, 13, 37], instead of traditional methods, have become one of the latest trends to predict airfare ticket prices.

This paper proposes a novel strategy for predicting air ticket prices based on the multi-attribute dual-stage attention (MADA) mechanism to address this problem. Besides, the Seq2Seq neural network is adopted to encode and decode the input multi-dimensional fare-related attributes. Moreover, dual-stage attention mechanisms [31] are employed to extract effective information variables. The mean square error loss function is used to train the real data to obtain the trend of fare changes.

Our main contributions are threefold.

1. An improved multi-attribute dual-stage attention mechanism model is proposed. The first attention mechanism is performed by the encoder in the input time series, which selects important weight information for the decoder layer. Subsequently, the decoder layer uses such weight information in its temporal attention mechanism to produce the final prediction outputs.
2. Various major models for airfare prediction were compared on real data sets. The results showed that the MADA model outperformed others in MSE, RMSE, and MAE indicators.
3. Finally, the accuracy of civil aviation ticket price prediction was compared among different prediction models, and the influence extents of different data attributes when the proposed model was in different hidden layers were also analyzed. In terms of RMSE, MSE, and MAE indicators, the MADA model outperformed the variant and benchmark models.

The rest of the paper is organized as follows. Section 2 introduces the related previous works about civil aviation fare prediction, and Section 3 introduces the relevant models, including a detailed description of model data preprocessing and the network models. Section 4 describes the algorithm in this paper. Subsequently, the experimental evaluations, including data sets, evaluation indexes, comparisons of experimental results, and ablation analysis, are illustrated in Section 5. Finally, in Section 6, the work in this paper and the direction for future research are summarized.

## Related work

Airfare prediction is essentially a time series forecast. The Auto-Regressive Integrated Moving Average (ARIMA) method is a traditional method for time series prediction, where AR and MA eliminate positive and negative correlations, respectively. AR and MA offset each other, and they usually contain two elements that can avoid overfitting. Gordiievych et al. [12] proposed to use the ARIMA model to build a system that will help customers make purchase decisions by predicting the price of air tickets. Besides, another idea called Facebook-prophet [35] is similar to STL (Seasonal-Trend decomposition procedure based on Loess) decomposition, which can divide the time-series signals into seasonal, trend, and residue ones. STL decomposition, as the name suggests, is better suited to dealing with seasonal time series data than traditional time series models in terms of control and interpretability.

In Random forest (RF), multiple decision trees are integrated by ensemble learning. XGBoost (Extreme Gradient Boosting Decision Tree) [5] is a machine learning algorithm with higher robustness and efficiency, which can be applied to detect problems and predict time series. To aid the consumer decision-making process, Wohlfarth et al. [7] integrated in the early phases of the cluster and used a variety of the latest supervised learning algorithms (classification tree (CART) and RF). They then utilize CART to understand meaningful rules and RF to provide information about each function’s relevance. To compare relative values with the total average price, Ren et al. [32] proposed utilizing LR, Naive Bayes, Softmax regression, and SVMs to develop a prediction model and categorize the ticket price into five bins (60% to 80%, 80% to 100%, 100% to 120%, and so on). The models were built using over 9,000 data points, comprising six features (such as the start of the departure week, the date of the price quote, the number of stops in the itinerary, and etc). Using the LR model, the authors reported the best training error rate of around 22.9%. Instead, the prices were classified as “higher” or “lower” than the average using an SVM classification model.

It has been used to predict crude oil prices [39] and housing prices [29], and it is more effective compared to with the traditional ARIMA. As the XGBoost and deep learning techniques continue to develop, researches about flight delays [13] have also integrated relevant random tree models with deep learning. The flight delays experiment shows LSTM cell is an effective structure to handle time sequences and random forest-based method can obtain good performance for the classification accuracy (90.2% for the binary classification) and overcome the overfitting problem.

As the deep learning technique continues to develop, RNN (LSTM, GRU) time series analysis and CNN+ *RNN* + attention prediction have been proposed as two prediction methods. Specifically, CNN captures short-term local dependence, while RNN captures long-term macro dependence. Attention is weighted for essential periods or variables, and the representative models for such strategy are TPA-LSTM [33], LSTNet [22] and MTA-RNN [9]. Shih et al. [33] introduce a different attention mechanism for selecting important time series and multivariate forecasting using frequency domain information. The attention dimension was allowed to be feature-wise by the authors in order for the model to learn interdependencies among various variables not only within the same time step, but also over all past times and series. Meanwhile, Guokun et al. [22] also made a model to predict the time-series, which through a combination of neural networks and recursive convolution advantage of neural networks as well as auto-regressive component, to form a better robustness of the model. Deep learning in passenger load factors and ticket prices, as well as a multi-granularity temporal attention mechanism RNN structure [9], has been reported to predict the relationship between ticket prices.

Besides, Seq2Seq can also predict time series, and it is combined with the attention mechanisms, such as DA-RNN [31], which adopts the dual-stage attention mechanism. In this way, the attention mechanism for time series is added in both the encoder and decoder layers in Seq2Seq. Experiments in this DA-RNN prove that increasing the temporal attention mechanism can capture more relevant input features. Another price forecast model based on the attention mechanism, TADA [6], has also been proposed and proved to produce more evident effects than a single-layer encoder with attention. The TADA splits influencing factors into internal and exterior features, and then uses the dual attention mechanism to forecast future price trends.

At present, although deep learning has received a lot of attention for stock forecasting [4, 16, 24, 27], it has received very little attention for forecasting civil aviation ticket prices. The RNN is the most widely used deep learning model for prediction, and it has gained a lot of traction. Recent years have seen a surge in approaches that use neural network structures to make the prediction results more accurate [17, 21, 38].

A summary for the discussed ticket price prediction models and correlation time series forecasting models is shown in Table 1.

**Table 1:** Summary of airline fare prediction models and correlation time series forecasting models

Category	Ref.	Addressed problem	Method	Performance result
	Tim et al. [18]	Predict the lowest ticket price before departure	Linear quantile mixed regression model	Short-term performance is reasonable, but long-term performance is inefficient.
	Tziridis et al. [28]	Find the optimal fare prediction model from the regression algorithm.	Eight regression machine learning models	Bagging Regression:87.42% accuracy and Random Forest Regression Tree:85.91%.
	Gordiievych et al. [12]	Prediction whether the price of ticket drops in the future.	ARIMA	Not given
Machine Learing	Wohlfarth et al. [7]	Predict the best time to buy tickets	CART and RF	CART and RF should be used for preregistered purchase periods to give a first coarse advice to the customer.
	Ren et al. [32]	Predict the lowest ticket price before departure	Ensemble model that uses LR, Naive Bayes, Softmax Regression, and SVM.	The training error of Naive Bayes and Softmax Regression reduced to 24.88% and 20.22% .SVM is also reduced by approximately 1%.
	GuanGui et al. [13]	Flight delay prediction	Ensemble model that relevant random tree models with deep learning	LSTM is capable of handling the obtained aviation sequence data, RF (90.2% for the binary classification) can overcome the overfitting problem.
	Guokun et al. [22]	Multivariate time series prediction	Deep learning: CNN and RNN to extract short-term local dependency patterns among variables.	The results show that three of the four experimental data sets have the best performance.
	Shih et al. [33]	Multivariate time series prediction	Deep learning: Attention mechanism for selecting important time series and multivariate forecasting using frequency domain information.	The proposed TPA-LSTM performs best in experiments.
Deep Learning	Deng et al. [9]	Prediction of flight passenger load factors.	Deep learning: CNN using multi-granularity temporal attention mechanism(MTA-RNN).	The proposed MTA-RNN performs best in experiments.
	YaoQin et al. [9]	Stock time series prediction	Deep learning: Dual-stage attention-based RNN.	The DA-RNN model has the best performance in the data set SML 2010 and NASDAQ 100 compared with other models.
	TongChen et al. [6]	Forecast sales volume in real-life commercial scenario.	Deep learning: Dual-stage attention-based RNN trend alignment with dual-attention, multi-task RNNs for sales prediction.	The results show that TADA prediction result is the best.

## The airfare prediction model

In this section,we first introduce the related work of data preprocessing and then we present the technical details of our proposed model MADA. Simultaneously, mathematically formulate the definition of civil airline fare prediction.

### Data preprocessing and profiling

**Fig. 1:**
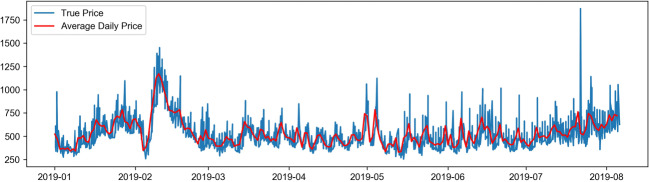
The trend of ticket price changes for a specific flight

**Fig. 2:**
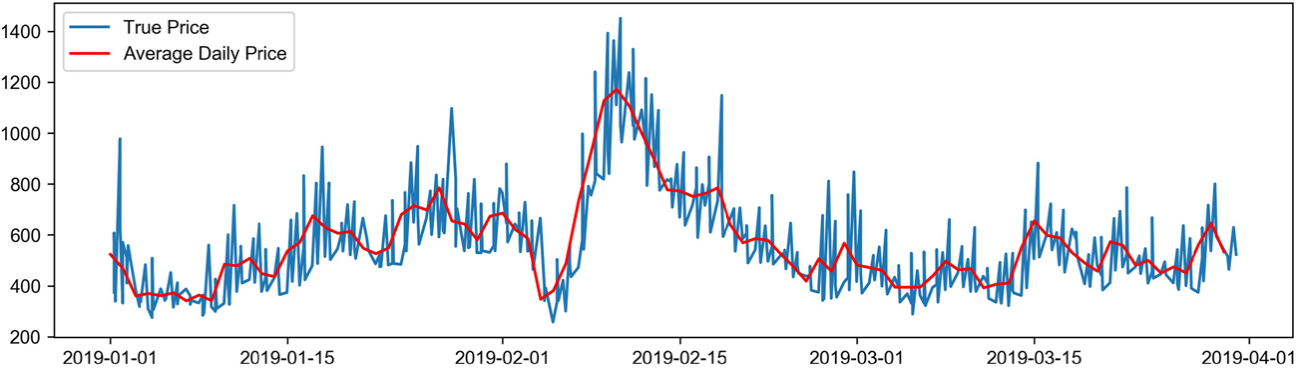
Trends of fare changes in the first quarter

**Fig. 3:**
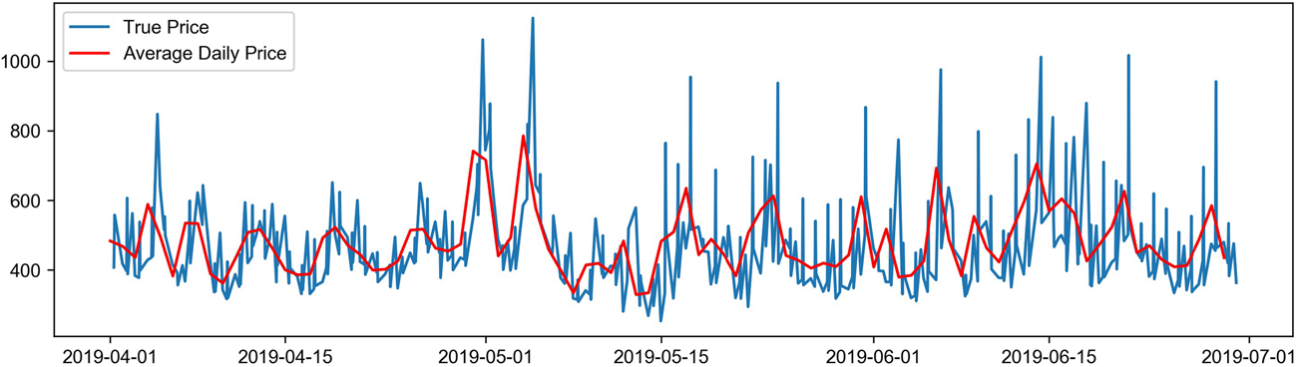
Trends of fare changes in the second quarter

**Fig. 4:**
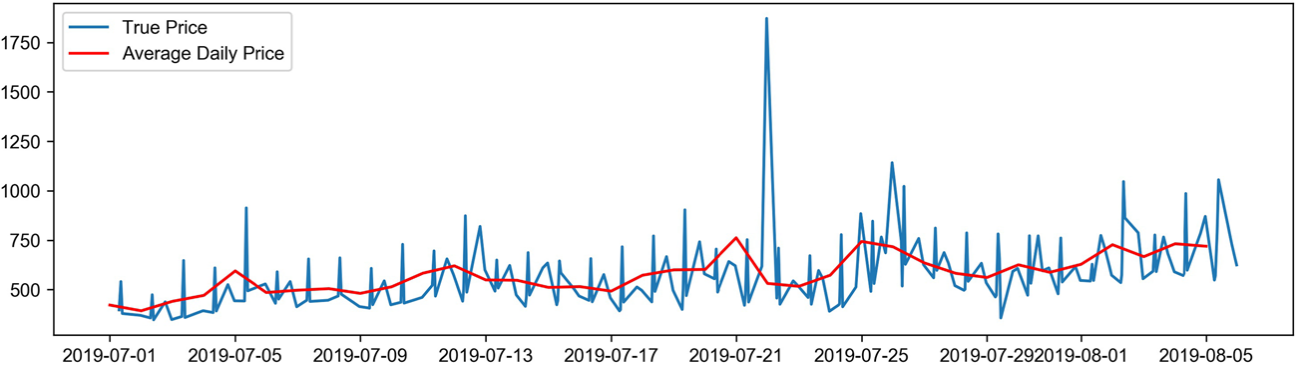
Trend of fare changes in the third quarter

Firstly, the data is preprocessed, and the missing values within the data are removed. Based on the currency exchange rates, the face value of ticket prices is represented as Chinese yuan, and the non-numerical data is vectorized to meet the requirements for data input into the neural network. Subsequently, identical route data is selected for model training. The attributes related to airfare prediction include departure airport (Dpt_AirPt_Cd), arrival airport (Arrv_Airpt_Cd), boarding time (Datetime), route (Air_route), flight number (Flt_nbr), airline (Airln_cd), the total number of guests (Pax_Qty_y), and fare (FARE) (Table 2). Finally, the flight ticket price data is visualized, as is shown in Fig. 1. Figures 2, 3 and 4 show the price changes in different quarters of the flight segment. For a specific flight, the red line represents its daily average price, and the blue line represents its real-time price changes. Note that only airfare information of the first eight months is displayed.

**Table 2:** Input attributes

Feature attribute	Description
Airln_cd	Airline
AirCrft_Typ	Aircraft type
Dpt_AirPt_Cd	Departure airfield
Arrv_Airpt_Cd	Arrival airport
Air_route	Route
Flt_nbr	Flight no.
Flt_Schd_Dpt_Tm	Flight take-off time
Weekday	Weekly attributes
Holiday	Holiday
Pax_Qty_y	Number of flights
Fare	Air ticket price

Figure 1 shows the dynamic fluctuation of actual ticket prices in the first eight months of a certain line segment. Figures 2, 3 and 4 show the price fluctuation trends of 3 quarters in a year, respectively. Particularly, Fig. 2 stands out because of the phenomenal growth around mid-February, which is because mid-February coincides with the traditional Chinese Spring Festival in that year. In the second quarter of Fig. 3, the air ticket prices exhibit periodic changes; however, as can be seen in the third quarter of Fig. 4, the variation in air tickets becomes relatively stable. Therefore, it is fair to deduce that generally speaking, the airfare of this particular flight segment shows periodic fluctuations. At the beginning of the year, the ticket prices vary significantly because of the holidays, and then they exhibit periodic changes. After the middle of the year, which is the off-season period, the ticket prices of this segment reveal a stable increase.

In one execution session of the model, the original data is cleaned to remove duplicate or missing values, and a table with information such as departure time, airline, flight number, route, and guests’ number is created.

Subsequently, the non-numerical values are encoded by One-Hot and vectorized to serve as the inputs into the neural network. After that, in an 8:2 ratio, the data is divided into training and test data.

**Fig. 5:**
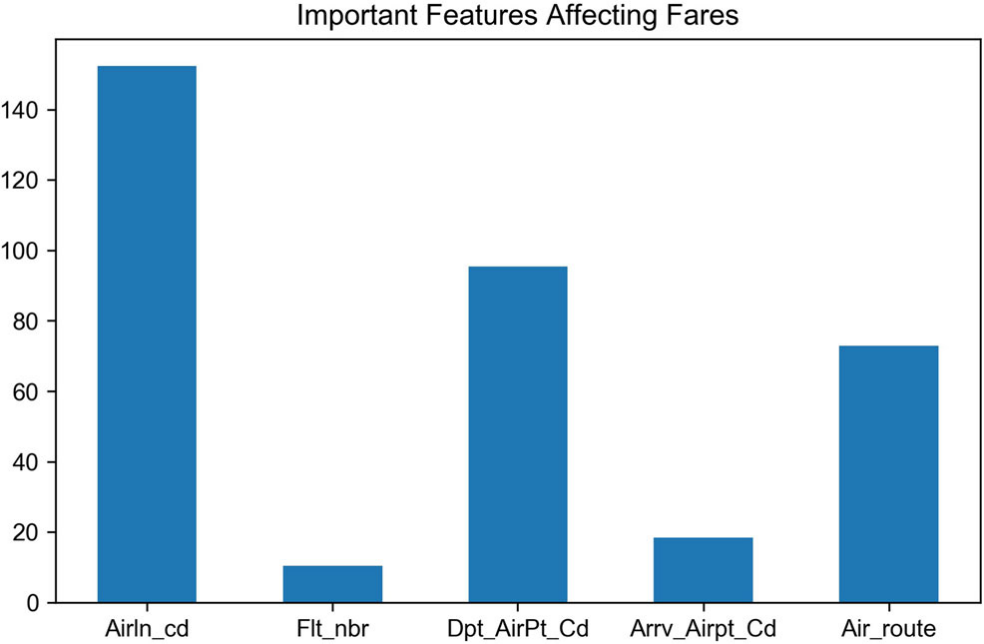
Important factors affecting fares

It’s worth noting that the selected feature attributes’ dimensionality is being reduced. First, properties that are relevant to flight fares are visualized (Fig. 5) with the feature selection module in the Scikit-Learn machine learning toolbox. From Fig. 5, it is obvious that airline (Airln_cd) is the most significant factor to affect ticket fares, followed by the departure airport (Dpt_AirPt_Cd) and the route (Air_route). By reducing the relatively non-critical dimensions, the model shows a higher overhead and generalization capability.

### The deep learning model for fare prediction

**Fig. 6:**
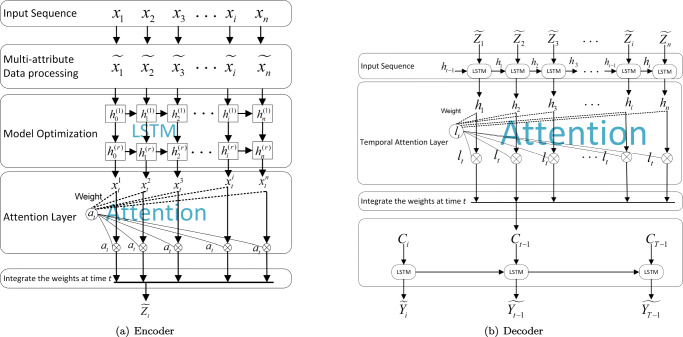
MADA Model

The structure of the MADA mechanism model proposed in this paper is shown in Fig. 6.

**(1) Network model**: To perform multi-dimensional airfare prediction, a multi-attribute dual-stage attention mechanism model is proposed in this paper.

Ticket price forecasts need multidimensional data, which are represented as *x*_1_,*x*_2_,…,*x*_*n*_. Then input *X* $= \left (x_{1},x_{2},\ldots ,x_{n}\right )^{T}=(x^{1},x^{2},\ldots ,x^{p})\in \ R^{n\ast p} $, where *p* represents the window size. The time periods *t* use *X* $= \left (x_{1},x_{2},\ldots ,x_{n}\right )^{T}=(x^{1},x^{2},\ldots ,x^{p})\in \ R^{n\ast p}$ to represent the processing results with multiple attributes. After that, *X* is input into the LSTM layer to obtain the feature vector, which is integrated with the feature weight *a*_*t*_ at time *t* to obtain the output $\widetilde {Z_{t}}$ in the encoder layer.

Next, the input for the decoder layer of the LSTM network is the time series $Z_{t}=(\widetilde {Z_{1}},\widetilde {Z_{2}},\widetilde {Z_{3}},...,\widetilde {Z_{n}})\ \in \ R^{n\ast p}$ of time *t* in the encoder layer. The decoding results are integrated with the feature weight *l*_*t*_ at time *t* to get the context vector *C*_*t*− 1_. Finally, the final predicted value $\widetilde {Y_{t-1}}$ is obtained from the final output layer in LSTM.

Following the steps above, the processed data can be input into the LSTM network for relevant trainings. The model employs a supervised learning methodology, with the multi-dimensional data (airline, flight number, departure airport, arrival airport, flight path, and flight number) representing *X* and the ticket price data representing *Y*. In this way, model training is enabled, and the MADA model can remember the law of changes of the relevant data. During the model training, various parameters need to be adjusted to optimize the model, so that both the training data and the test data can achieve the best possible results.

**(2) Loss function**: The mean square error MSELoss is used as the loss function. Set the vectors *s* and *y* as the predicted and actual values, respectively. MSELoss calculates the error (scalar *e*) and the gradient of *e* concerning *s*.

$$ e=M S E \operatorname{Los} s(s, y)=\frac{1}{n} {\sum}_{t=1}^{n}\left( s_{t}-y_{t}\right)^{2} $$

The solution is:

$$ \begin{array}{c} \frac{d e}{d s}=\frac{2}{n}\left( \left( s_{1}-y_{1}\right),\left( s_{2}-y_{2}\right), \ldots,\left( s_{n}-y_{n}\right)\right) \\ \frac{d e}{d y}=-\frac{d e}{d s} \end{array} $$

The mean square error loss is calculated by the distance between the target and the calculated values, and the gradient of each step is obtained by backward propagation. After multiple iterations, the minimum loss is obtained.

**(3) Activation function**: The Leaky ReLU [26] and Softmax are used as the activation functions. Particularly, the Leaky ReLU can extract the feature information hidden in the data and map it to the corresponding ranges. The equation of the Leaky ReLU function is:

1 $$ y_{i}=\left\{\begin{array}{ll} x_{i}, & \text { if } x \geq 0 \\ \frac{x_{i}}{a_{i}}, & \text { if } x<0 \end{array}\right. $$

Compared with the ReLU [11], a general activation function, the Leaky ReLU is used in this paper because it can reasonably divide the negative values. Empirically, the Leaky ReLU is more efficient than the ReLU.

On the other hand, Softmax can convert all the input values into others within the range of 0–1. Its equation is:

2 $$ y_{i}=S(z)_{i}=\frac{e^{z_{i}}}{{\sum}_{j=1}^{C} e^{z_{j}}}, i=1, \ldots, C $$

*Z* is the output of the previous layer, which serves as the input of Softmax. The predicted object’s dimension is *C*, and *y*_*i*_ is the probability that it belongs to the *C*-th category.

**(4) Optimizer**: Adam (Adaptive Moment Estimation) [20] is a first-order optimization algorithm that can be used instead of the conventional stochastic gradient descent. Furthermore, iterations based on the training data can be used to adjust neural network weights. Adam is essentially RMSprop with a momentum term, which uses the first-order and second-order moment estimations of the gradient to realize dynamic adjustments of the learning rate for each parameter. It is especially beneficial because, after bias correction, the learning rate at each iteration has a defined range, resulting in reasonably stable parameters. Its equations are as follows:

3 $$ m_{t}=\mu * m_{t-1}+(1+\mu) * g_{t} $$

4 $$ n_{t}=v * n_{t-1}+(1-v) * {g_{t}^{2}} $$

5 $$ \widehat{m_{t}}=\frac{m_{t}}{1-\mu^{t}} $$

6 $$ \widehat{n_{t}}=\frac{n_{t}}{1-v^{t}} $$

7 $$ {\varDelta} \theta_{\mathrm{t}}=-\frac{\widehat{\mathrm{m}_{\mathrm{t}}}}{\sqrt{\mathrm{n}_{\mathrm{t}}+\epsilon}} * \eta $$

The letter meaning in the above formulas are as follows :*μ*,*v* ∈ [0,1] represents exponential decay rates for the moment estimates. *m*_0_ initialize first-order moment vector, *n*_0_ initialize second-order moment vector, *𝜃*_0_ initialize parameter vector, *t* initialize timestep, and *η* is stepsize.

Among them, (3) and (4) are the first-order and second-order moment estimations of the gradient, which can be considered the expected estimations of *E*|*gt*| and *E*|*gt*^2^|, respectively. Besides, (5) and (6) are two correction equations to (3) and (4), so that they can be approximated as unbiased estimates of expectations. The direct moment estimations of the gradients, based on the equations, do not require additional memory and can be dynamically adjusted according to the gradients. The last item’s front part is a dynamic constraint on the learning rate *n* that is located within a precise range.

### Dual-stage attention mechanism

Aside from the Seq2Seq model, the model also integrates the attention mechanism into its time series dimension in its encoder and decoder layers.

#### Encoder with input attention

The input time series is *X* = (*x*^1^,*x*^2^,…,*x*^*p*^) = (*x*_1_,*x*_2_,…,*x*_*n*_)^*T*^ ∈ *R*^(*n*∗*p*)^, where *n* is the window of the input time series, **h**_*t*_ ∈ *R*^*m*^ represents the encoder layer’s hidden state at time *t*, and *m* represents the hidden state.

8 $$ \mathbf{h}_{t}=LSTM\left( \mathbf{h}_{t-1}, \mathbf{x}_{t}\right) $$

LSTM is used as the encoder model. The forget gate, the input gate, and the output gate are all required gates for each LSTM. The output results are expressed as **h**_*t*_.

Where (**h**_*t*− 1_;**x**_*t*_) ∈ *R*^*m*+*n*^ is the result of concatenating the previously hidden layer **h**_*t*− 1_ with the current input **x**_*t*_. LSTM is chosen as the loop unit because it can evade gradient disappearance and explosion problems, enabling better capture of the time series data input.

Computer vision’s proposed attention mechanism will contribute to enhance the model’s prediction accuracy. Within the time series *X* = (*x*^1^,*x*^2^,…,*x*^*p*^) = (*x*_1_,*x*_2_,…,*x*_*n*_)^*T*^ ∈ *R*^(*n*∗*p*)^, deterministic attention is used to extract the input time dimensions. The previously hidden states, namely **h**_*t*− 1_ and **v**_*t*− 1_, serve as the attention input in the LSTM of the encoder layer. The equations are as follows:

9 $$ {b_{t}^{p}}=\mathbf{U}_{e}^{\top} \tanh \left( \mathbf{W}_{e}\left[\mathbf{h}_{t-1} ; \mathbf{v}_{t-1}\right]+\mathbf{B}_{e} \mathbf{x}^{p}\right) $$

and

10 $$ {\alpha_{t}^{p}}=\frac{\exp \left( {b_{t}^{p}}\right)}{{\sum}_{i=1}^{n} \exp \left( {b_{t}^{i}}\right)} $$

Among them, the parameters of **U**_*e*_ ∈ *R*^*T*^, **W**_*e*_ ∈ *R*^*T*∗2*m*^, and **B**_*e*_ ∈ *R*^*T*∗*T*^ must be learned, and the equation (9) should not be biased. The equation (10) calculates the attention at time *t*. Softmax is used to normalize the corresponding weights. Attention of this input data can extract the time series information for the decoder to perform subsequent steps. After that, the input time series can be extracted adaptively following the equation (11):

11 $$ \tilde{\mathbf{x}}_{t}=\left( {\alpha_{t}^{1}} {x_{t}^{1}}, {\alpha_{t}^{2}} {x_{t}^{2}}, \cdots, {\alpha_{t}^{n}} {x_{t}^{n}}\right)^{\top} $$

Then, using the equation (12), the hidden state at time *t* is updated:

12 $$ \mathbf{h}_{t}=LSTM\left( \mathbf{h}_{t-1}, \tilde{\mathbf{x}}_{t}\right) $$

**x**_*t*_ is replaced by $\tilde {\mathbf {x}}_{t}$, and the weight of the input time series is calculated.

#### Decoder with attention

To predict the final result $\tilde {\mathbf {Y}}_{T}$, both the encoder and the decoder are used. However, the model’s robustness in predicting time series data is lacking, particularly at longer input time series lengths. Therefore, the decoder adopts the temporal attention mechanism based on the concatenation of hidden state **d**_*t*− 1_ and the cell hidden state $\mathbf {v}_{t-1}^{\prime }$ of the previous encoder layer. Input to the time attention network in the decoder, such as **U**_*d*_, **W**_*d*_, and **B**_*d*_, need to be trained to learn parameters. In the equation (13), ${\beta _{t}^{i}}$ is the weight layer in the decoder, and work out at ${l_{t}^{i}}$ is the attention weight at time *t* by equation (14).

13 $$ {\beta_{t}^{i}}=\mathbf{U}_{d}^{\top} \tanh \left( \mathbf{W}_{d}\left[\mathbf{d}_{t-1} ; \mathbf{v}_{t-1}^{\prime}\right]+\mathbf{B}_{d} \mathbf{h}_{i}\right) $$

and

14 $$ {l_{t}^{i}} =\frac{\exp \left( {\beta_{t}^{i}}\right)}{{\sum}_{j=1}^{T} \exp \left( {\beta_{t}^{j}}\right)} $$

Each hidden **h**_*i*_ in the encoder layer is used as the input in the decoder layer and calculated with its corresponding attention weight to obtain a weighted average context vector **C**_*t*_. The total hidden input is [*h*_1_,*h*_2_,*h*_3_,...,*h*_*T*_].

15 $$ \mathbf{C}_{t}={\sum}_{i=1}^{T} {l_{t}^{i}} \mathbf{h}_{i} $$

**C**_*t*_ is the different times in the context vector. Once the total amount of context vectors is obtained, it is combined with the input target (*y*_1_,*y*_2_,...,*y*_*T*− 1_), and that gives us:

16 $$ \tilde{y}_{t-1}=\tilde{\mathbf{w}}^{\top}\left[y_{t-1} ; \mathbf{C}_{t-1}\right]+\tilde{b} $$

Where *y*_*t*− 1_ is the input of the decoder layer, **C**_*t*− 1_ is the context vector.

17 $$ \mathbf{d}_{t}=LSTM\left( \mathbf{d}_{t-1}, \tilde{y}_{t-1}\right) $$

Then $\tilde {y}_{t-1}$ and the input **d**_*t*− 1_ in the decoder layer are concatenated, and the concatenation results are input into the LSTM network to find out **d**_*t*_. Subsequently, **d**_*t*_ is concatenated with **C**_*t*_ in the fully connected neural network (FC), and $\tilde {\mathbf {Y}}_{T}$ is the final prediction result from training.

18 $$ \tilde{\mathbf{Y}}_{T}=FC\left( \mathbf{d}_{t}, \mathbf{C}_{t}\right) $$

And that concludes the introduction of the training process for the MADA model.

## Algorithms

The entire execution process of our MADA model is consisted of Algorithm 1 (Multi-attribute Data Processing) and Algorithm 2 (Multi-attribute Dual-stage Attention Mechanism model). 
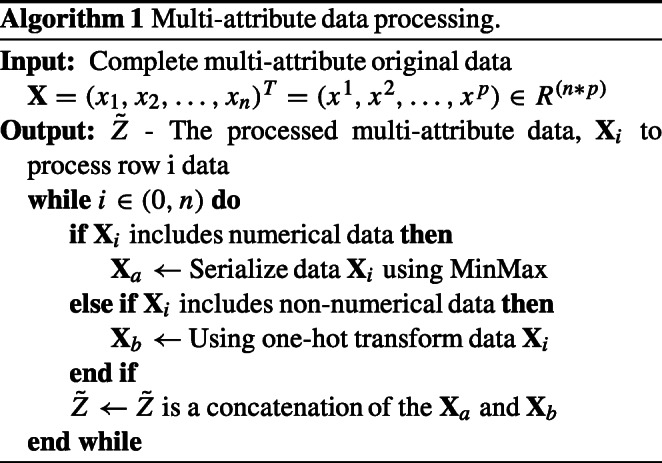


In Algorithm 1, the multi-attribute content *X* from the original user is entered to determine the data type. Before executing Algorithm 1, the attributes are preprocessed based on their types. For numerical data, MinMax is used to normalize them within the range of [-1,1]; when the data is non-numerical, it is encoded as a One-Hot number. Finally, after such preprocessing, the values are concatenated to obtain the output $\tilde {Z}$, which serves as the input for Algorithm 2.

In Algorithm 2, after inputting $\tilde {Z}$, to get the final prediction result $\tilde {\mathbf {Y}}_{T}$, **d**_*t*− 1_ and **v**_*t*− 1_ are fed into the decoder layer. MSELoss then calculates the difference between the real and expected values, and the learning parameters are updated by back propagation to gradually improve the model’s capability of generalization. 
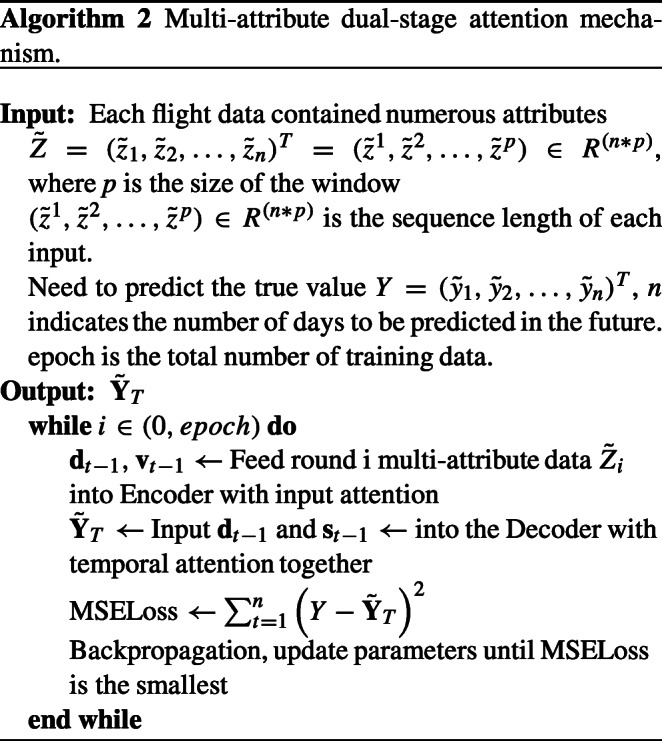


## Experimental evaluations

In this section, we conduct experiments on real civil aviation datasets to showcase the advantage of MADA in the task of airline fare prediction.

### Datasets

For experimental evaluations, our model was implemented in Python 3.7.6 and performed on an Ubuntu 18.04 with a 2.5GHz Intel Core i7 CPU and 8GB memory. The data set was a two-year anonymous airfare record from a real airline, and the training set contained more than 1.7 million data pieces, including essential attributes such as airline, flight number, departure time, and passenger volume. The data set contains more than 200 routes, and the paper selects one of the representative routes for discussion.

### Evaluation metrics

The absolute error (MSE), root mean square error (RMSE) and mean absolute error (MAE) were applied as the assessment metrics. Their calculation equations are:

$$ \text{MSE}=\frac{1}{m} {\sum}_{i=1}^{m}\left( y_{\text {test}}^{(i)}-\hat{y}_{\text {test}}^{(i)}\right)^{2} $$

$$ \text{RMSE}=\sqrt{\frac{1}{m} {\sum}_{i=1}^{m}\left( y_{\text {test}}^{(i)}-\hat{y}_{\text {test}}^{(i)}\right)^{2}} $$

$$ \text{MAE}=\frac{1}{m} {\sum}_{i=1}^{m}\left|y_{\text {test}}^{(i)}-\hat{y}_{\text {test}}^{(i)}\right| $$

According to the predicted label, the reference values of these three metrics help train the model for better generalization capability.

### Baselines

ARIMA, RF, XGBoost, CNN-LSTM, CNN-LSTM + Attention, and other mechanical models were used in this paper for comparison with the MADA prediction model. ARIMA, RF, and XGBoost were executed without any further configurations.

#### LSTM-CNN [15]

This approach first uses the LSTM model, followed by CNN for parameter classification. Initially, this model was used to predict gold prices.

#### CNN-LSTM [14]

This model uses CNN first, and then connects to LSTM. These two sub-modules are combined to form a CNN-LSTM model. For time series forecasting, the CNN-model is frequently used.

#### CNN-LSTM+Attn [25]

In this model, CNN is used to extract multidimensional attributes, and the results are entered into LSTM, then the final results are output through the attention mechanism. The model is used to predict PM2.5 concentration.

#### Seq2Seq [34]

Seq2Seq is first proposed to be used to deal with language translation problems. This paper extends its application to predict time series problems.

#### Seq2Seq+Attention [3]

Seq2Seq with attention is first proposed to be used to deal with language translation problems when the input sequence length is longer. This paper extends its application to predict time series problems.

#### MADA

The model proposed in this paper adopted multi-attribute data preprocessing, and the attention mechanism was added to both the encoder and the decoder layers.

### Experimental results

Generally speaking, the new MADA model produced significantly lower MSE, RMSE, and MAE results than the previous models, implying that it can better predict price fluctuation trends. The experiment applied predominantly the Pytorch deep learning framework for extensive comparisons in terms of airfare forecasts and analyses. With repeated parameter adjustments, an optimal model was trained with Adam of unequal steps until the model parameters converged. During the experiments, one of the routes’ fare is used to compare the prediction results produced by the involved models effectively.

Table 3 shows the data comparison results when the time window is *T*= 30 for a 7-day forecast (*n*= 7 days).

#### Performance comparison

The performance comparison results can be seen in Table 3, in which the indicators for comparison are MSE, RMSE, and MAE. Judging from the experimental results, the predictions produced by the MADA model were significantly more preferred with much lower MSE, RMSE, and MAE than those obtained from traditional machine learning. Particularly, it is noteworthy that the MADA model integrated multi-dimensional data prediction, and it showed greater relevant input attributes, suggesting more accurate data prediction.

**Table 3:** Comparison of MADA and various models

Model	MSE	RMSE	MAE
ARIMA [2]	0.286090 ± 0.003	0.534880 ± 0.04	0.461500 ± 0.004
RF [36]	0.03893 ± 00.002	0.19730 ± 0.003	0.14092 ± 0.003
XGBoost [8]	0.00973 ± 0.001	0.09865 ± 0.002	0.09365 ± 0.004
LSTM-CNN [15]	0.08562 ± 0.003	0.29261 ± 0.003	0.16951 ± 0.005
CNN-LSTM [14]	0.07019 ± 0.001	0.26493 ± 0.002	0.13326 ± 0.003
CNN-LSTM + Attn [25]	0.07812 ± 0.001	0.27950 ± 0.003	0.12005 ± 0.004
Seq2Seq [34]	0.00028 ± 0.0002	0.01663 ± 0.002	0.01161 ± 0.003
Seq2Seq+Attn [3]	0.0011 ± 0.0002	0.03390 ± 0.005	0.02544 ± 0.005
**MADA**	**0.00022 ± 0.0002**	**0.01489 ± 0.003**	**0.01134 ± 0.004**

Bold entries signify the model proposed in this article

Moreover, the MADA mechanism is more effective in terms of extracting time series than common deep learning models. However, it took more time to train a MADA model, and the prediction results still need much tuning for greatest accuracy. In short, the MADA model proposed in this paper is more preferable than other methods in predicting air ticket prices.

#### Effectiveness comparison

**Fig. 7:**
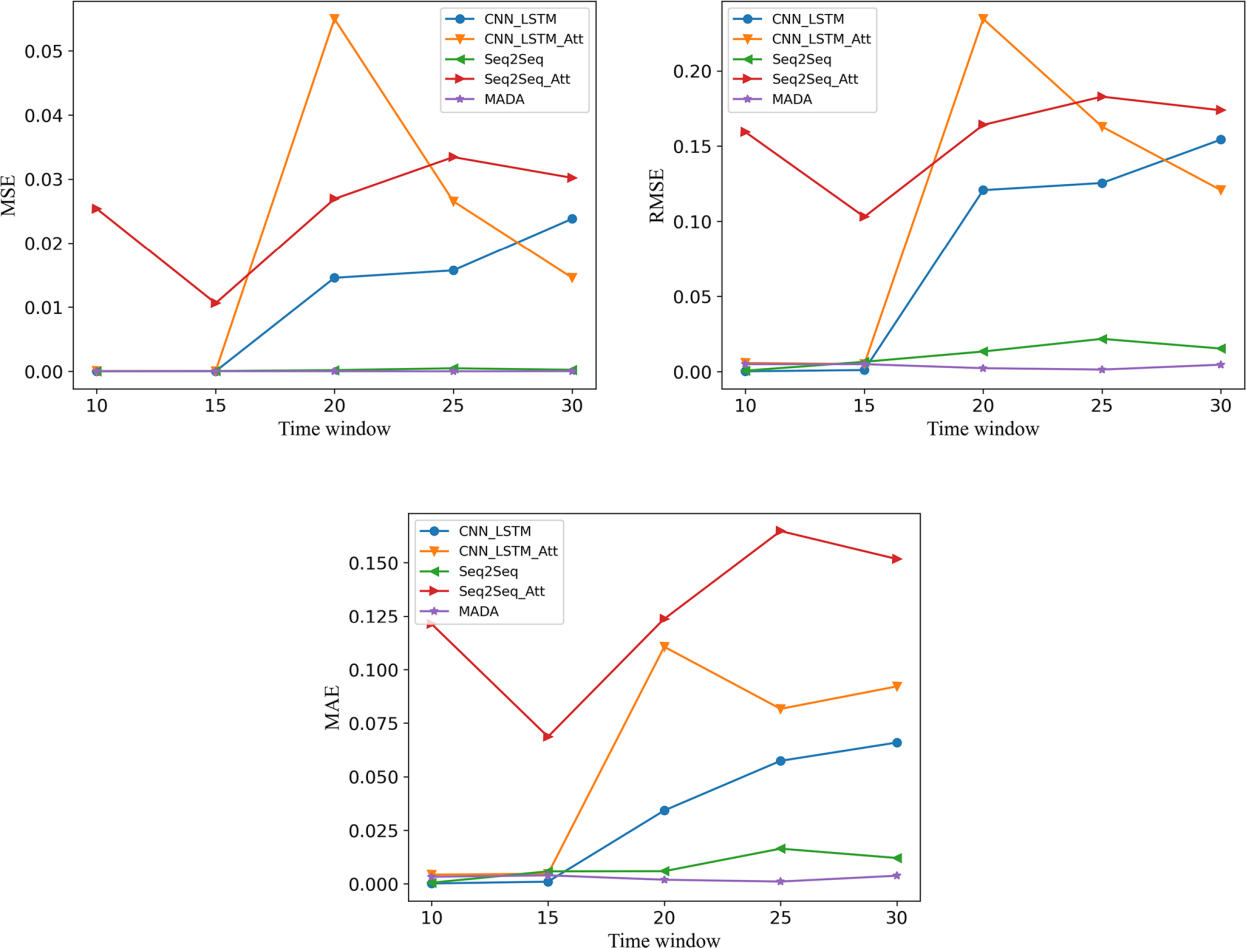
Evaluation index of different models

To compare the prediction effects, data from a certain flight was used for forecasts. For data preprocessing, models such as CNN-LSTM, Seq2Seq, and MADA were used for prediction. Figure 7 shows the fluctuation trends of MSE, RMSE, and MAE predicted by the three models.

The X-axis in Fig. 7 indicates that different time windows were used, and the data in the table was used to predict the prices next day under different time windows, which were 10, 15, 20, 25, and 30 days. Under different time windows, other models, such as CNN-LSTM + Attn, all became unstable in their performance. However, the MADA revealed better stability and more preferable performance under different time windows.

Meanwhile, based on the experimental results, if the model wants to predict the fare next day, the data from the past 15 days would be necessary for best training.

**Fig. 8:**
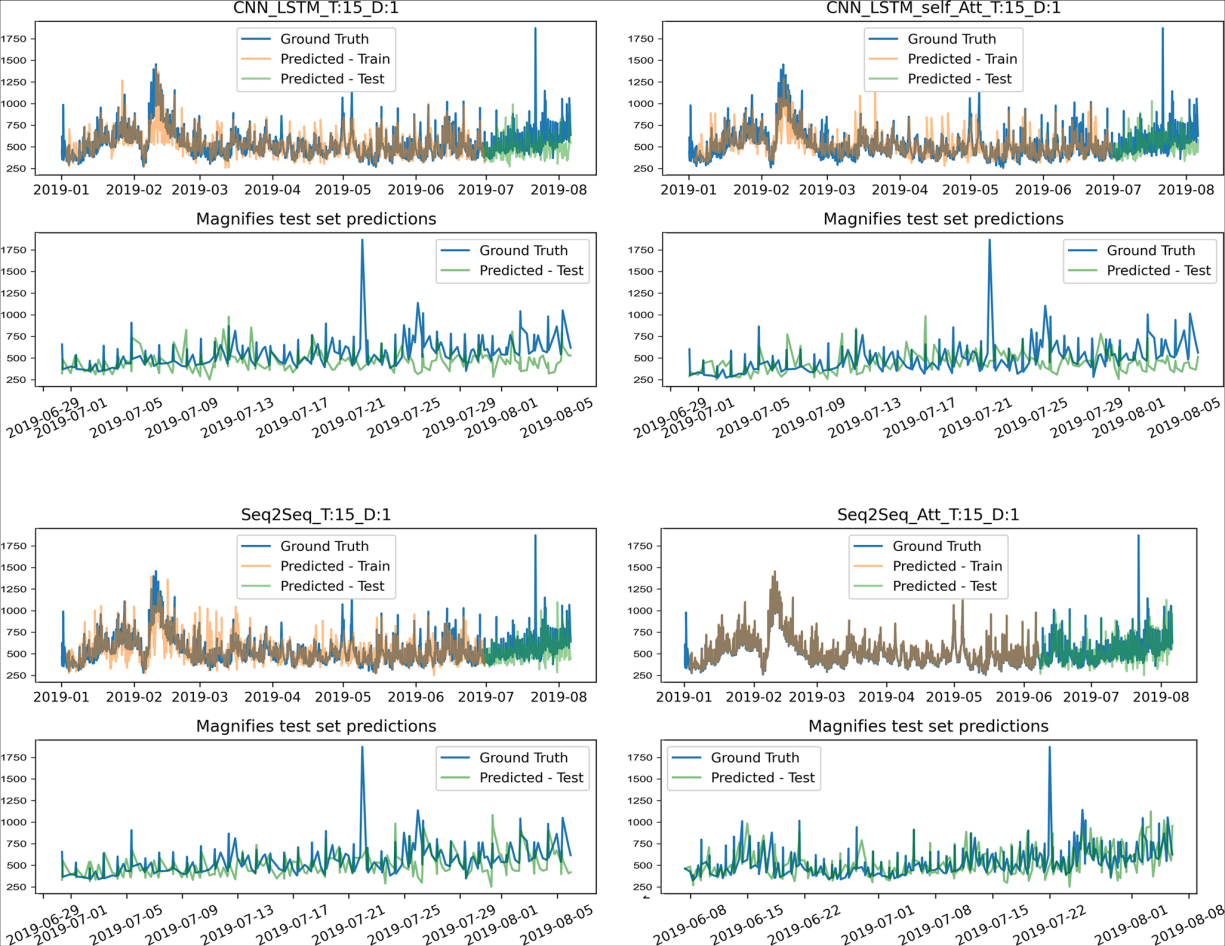
Visual comparison of different models, where T represents the time width, and D represents the number of days predicted in the future

**Fig. 9:**
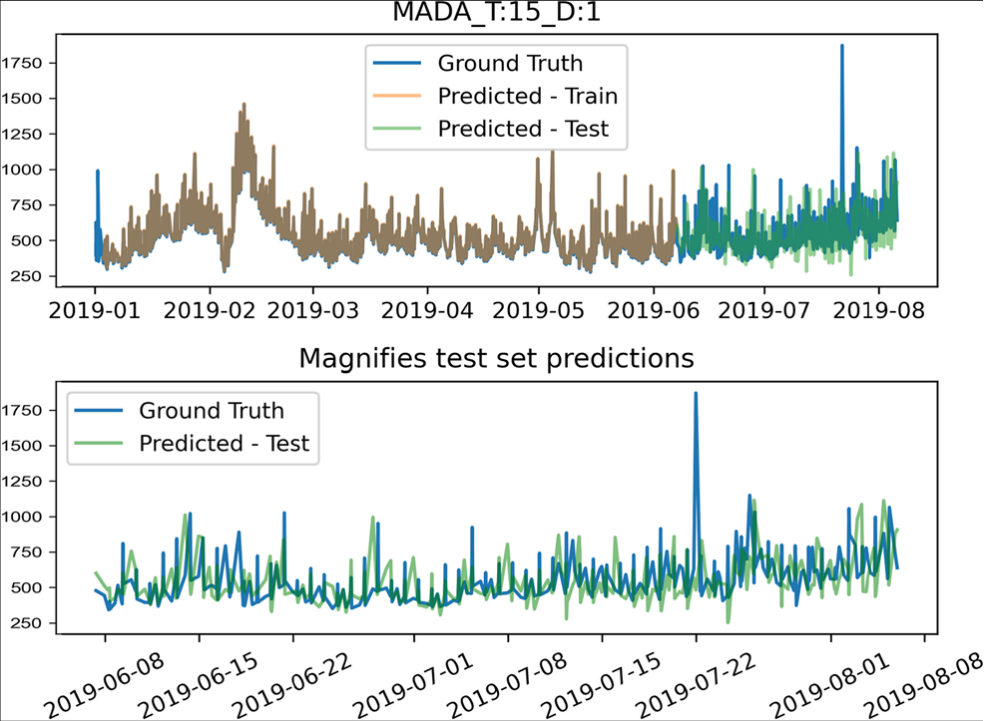
Visual comparison of different models, where T represents the time width, and D represents the number of days predicted in the future

In Fig. 8, the predictions from CNN-LSTM, CNN-LSTM + Attn, Seq2Seq, and Seq2Seq + Attn were compared. Figure 9 depicts the MADA model’s visualization results. Among them, the effects of the training set and the test set on the final predictions are shown in the upper parts of the graphs, while the effect of visualizing the upper half of the test set on the final predictions is shown in the lower parts. It can be concluded from the graphs that the MADA model produces more advantageous results in terms of civil aviation fare prediction.

#### Ablation study

The ablation study involved experiments on the proposed model MADA with different structures. The original model was altered as the follows.

##### MADA_nAttn

Encoders and decoders with 16, 32, 64, 100, or 128 hidden layers were used to build the model. The model structure used in the encoders and decoders was LSTM;

##### MADA_sAttn

Seq2Seq with 16, 32, 64, 100, or 128 hidden layers were used to build the model, followed by the addition of the temporal attention mechanism to the decoder layer;

##### MADA

Seq2Seq with 16, 32, 64, 100, or 128 hidden layers were used. Subsequently, the temporal attention mechanism was added to the encoder and decoder layers to build the model.

**Fig. 10:**
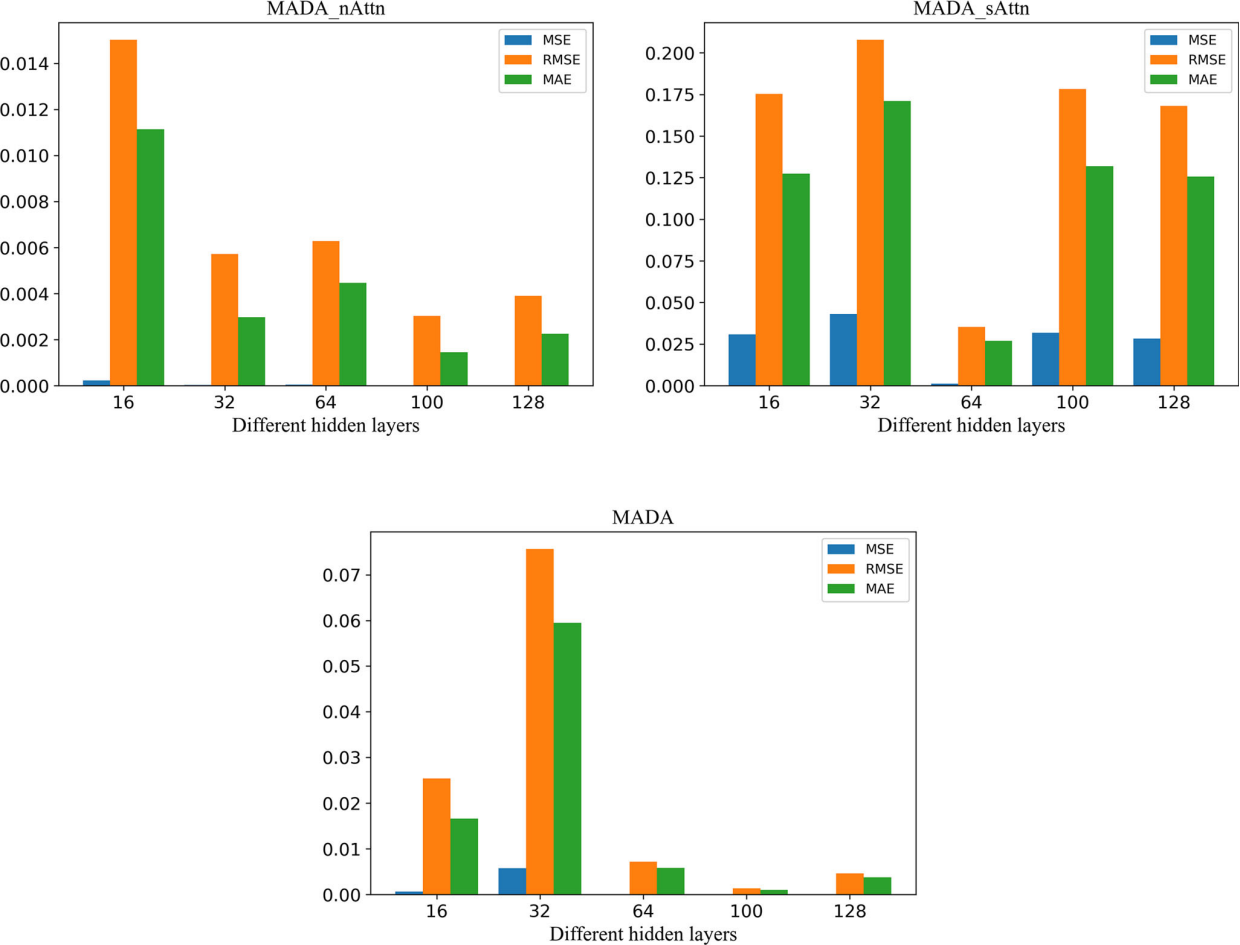
Compare different hidden layer models

The experimental results are shown in Fig. 10, in which the MSE, RMSE, and MAE of the MADA_nAttn model were negatively correlated with the number of hidden layers. Besides, the prediction results from the MADA_nAttn model became more favorable as the number of hidden layers rose. However, after producing the optimal results when there was 64 hidden layers, MADA_nAttn would lead to less satisfactory results when the number of model layers continued to increase. This is because the model does not perform well in the data set, and the hidden information within the data cannot be learned from a single attention, and such inaccessibility was exacerbated by a growing number of hidden layers. On the other hand, the MADA model proposed in this paper cannot learn the hidden information in the data with a small number of hidden layers (no larger than 32). However, as the number of hidden layers grew, the MADA model outperforms other deep learning models.

Besides, the importance of multidimensional attributes has been studied. Different attributes were input into the MADA model for training, and the prediction conditions were set as the time window *T*= 15 and *n*= 1. Here, the following different variants were defined.

##### MADA_nEx

There were no weekend or holiday attributes in the multi-attribute data.

##### MADA_nAttn_allData

The data entered to the model was complete, but no attention mechanism was added.

##### MADA_sAttn_allData

The data entered by the model was complete, but only temporal attention was added to the decoder layer.

Table 4 compares the MADA variant models by different indicators, and only the average result was taken.

**Table 4:** Comparison of the variant MADA models

Model	RMSE	MSE	MAE
MADA_nEx	0.00976	0.00010	0.00753
MADA_nAttn_allData	0.02422	0.00059	0.01768
MADA_sAttn_allData	0.01771	0.00031	0.0146
**MADA**	**0.00610**	**0.00004**	**0.00463**

Bold entries signify the model proposed in this article

It can be seen from Table 4 that MADA_nEx produced results that were less accurate than the original MADA, indicating that multi-attribute data input plays a vital role in the prediction accuracy. Besides, MADA_sAttn was more accurate in prediction compared to MADA_nAttn, suggesting the relevance of the attention mechanism in prediction.

## Conclusion

Currently, the prediction for civil aviation ticket prices remains rather inaccurate and unreliable. To solve such problem, a prediction method based on MADA is proposed.

Judging from the experimental results, the MADA-based method can produce more accurate prediction results than the traditional methods for civil aviation ticket prices. Moreover, with multidimensional training models, the prediction results will be more accurate. Combined with the dual-stage attention mechanism, the implicit information of time series can be extracted to the utmost extent.

Although MADA has a certain effect from the experimental results, there are still some problems based on the current research. Specifically, first of all, ticket prices will change with other uncontrollable attributes, such as weather conditions will also affect the change in ticket prices. Secondly, although this paper to do a lot more research in airlines fare prediction, but optimal purchase time prediction has not been studied. The prediction of the best time to buy air tickets may become a research direction in this field next. In addition, as far as airlines are concerned, there are also issues such as demand prediction and price discrimination that require further in-depth research.

In the future, more accurate prediction methods should be explored to optimize the current imperfections. The prediction of civil aviation ticket prices can be realized by deep learning methods, so that the ticket buyers can choose a more reasonable period to purchase. At the same time, the company can also increase its corresponding revenue through predictive models. There is a tradeoff between money saving by customer and increasing revenue by companies. Therefore, there is a need for a prediction model that can predict the optimal ticket prices that can bring mutual benefit both for customers and airlines.
